# Solubility of mesalazine in pseudo-binary mixtures of choline chloride/ethylene glycol deep eutectic solvent and water at 293.15 K to 313.15 K

**DOI:** 10.1186/s13065-023-01064-4

**Published:** 2023-11-28

**Authors:** Elina Armani, Parisa Jafari, Salar Hemmati, Elaheh Rahimpour, Mohammad Barzegar-Jalali, Abolghasem Jouyban

**Affiliations:** 1 Student Research Committee, Faculty of Pharmacy, Ardabil University of Medical Sciences, Ardabil, Iran; 2 Pharmaceutical Analysis Research Center and Faculty of Pharmacy, Tabriz University of Medical Sciences, Tabriz, Iran; 3 Kimia Idea Pardaz Azarbayjan (KIPA) Science Based Company, Tabriz University of Medical Sciences, Tabriz, Iran; 4 Drug Applied Research Center, Tabriz University of Medical Sciences, Tabriz, Iran; 5 Infectious and Tropical Diseases Research Center, Tabriz University of Medical Sciences, Tabriz, Iran; 6 Research Center for Pharmaceutical Nanotechnology, Tabriz University of Medical Sciences, Tabriz, Iran; 7 Biotechnology Research Center, Tabriz University of Medical Sciences, Tabriz, Iran; 8 Pharmaceutical Sciences Research Center, Shahid Beheshti University of Medical Sciences, Tehran, Iran

**Keywords:** Mesalazine solubility, Choline chloride/ethylene glycol deep eutectic solvent, Solution thermodynamics, Cosolvency models, Enthalpy-entropy compensation analysis

## Abstract

Mesalazine (5-ASA) is a medication utilized to treat inflammatory bowel diseases involving ulcerative colitis and Crohn’s disease. Mesalazine has fewer side effects but the low solubility and bioavailability of it is responsible for its delayed onset of action. Hence, the goal of this study is to determine the molar solubility of 5-ASA in aqueous pseudo-binary mixtures containing low toxic biocompatible choline chloride/ethylene glycol deep eutectic solvent (ChCl/EG DES) with DES mass fraction of 0.0–1.0 using a shake-flask technique at 293.2–313.2 K and approximately 85 kPa. The experimental results indicated that the solubility of 5-ASA enhanced by addition of DES mass fraction and also increasing temperature. The molarity values of 5-ASA were then modelled by some traditional cosolvency models, and regressed each model parameters. The back-computed molarity of 5-ASA using the selected cosolvency models presented a good consistency with the experimental data (lower mean percentage deviation than 5.14%). Moreover, the Gibbs and van’t Hoff equations were employed to compute the thermodynamic functions of 5-ASA dissolution process in ChCl/EG DES + water from the temperature dependency of solubility data. This analysis presented an endothermic and entropy-driven process of 5-ASA dissolution in ChCl/EG DES + water. Furthermore, enthalpy-entropy compensation analysis represented non-linear enthalpy dissolution *vs*. Gibbs free energy compensation plots with positive and negative slopes for 5-ASA whereas the positive and negative slopes were probably due to the enhance in solvation of 5-ASA by ChCl/EG DES molecules and the solvent-structure loosing, respectively.

## Introduction

Mesalazine (mesalamine or 5-aminosalicyclic acid) is an aminosalicylate drug applied for treating mild to moderate inflammatory bowel disease involving active ulcerative colitis and Crohn's disease along with also to maintain remission once achieved [1]. Mesalazine (5-ASA) can also act as an intermediate drug for synthesizing some drugs [2]. This drug as a class IV drug has a low solubility in water [3] which affected its bioavailability and absorption and so the improvement its solubility in water has more importance in the pharmaceutical industry. It is known that measuring drug solubility in different aqueous and non-aqueous solvent mixtures can provide some useful information regarding the purifying raw drug in the production procedure and pre-formulation investigations [4]. In the literature, the solubility of 5-ASA has been increased by various ways including salt formation, pH adjustments [5], solid dispersion [6], and cosolvents [7, 8]. Among these methods, cosolvency is a traditional way to increment the drug solubility [9].

To date, the solubility profile of 5-ASA has been reported in different binary mixtures including propylene glycol (PG), polyethylene glycol 400 (PEG 400), ethanol, 1-or 2-propanol, acetonitrile, ethylene glycol (EG), polyethylene glycol dimethyl ether 250 (PEGDME 250) or N-methyl-2-pyrrolidone + water [4, 9–16], N-methyl-2-pyrrolidone, carbitol or PG + ethanol [17, 18]. However, some of these reported binary mixed solvents contain the volatile, high-toxic and costly organic solvents which has a negative effect on the environment. In this respect, the use of deep eutectic solvents (DESs) as a safer solvent (5th concept from green chemistry [19]) have been developed for the solubilization of some drugs [20, 21]. In the case of 5-ASA, its solubility in choline chloride/PG (ChCl/PG), betaine/PG, betaine/EG or betaine/glycerol DESs and their aqueous mixtures have investigated and reported an increase in solubility of 5-ASA in water after addition of DESs [20, 21]. Among the different H-bond acceptor used for the formation of DESs, ChCl is a cheap, renewable, non-toxic, and biodegradable quaternary ammonium salt and the corresponding DESs utilized broadly as a drug solubilization agent [20, 22–24]. As above-mentioned, the solubility of 5-ASA has been determined in neat ChCl/PG DES and its aqueous mixtures [20]; however, the high viscosity of this DES (72.10 mPa s at 298.15 K) [25] can be affected the drug solubilization in mixtures. For identifying the role of ChCl-based DESs viscosity on their solubilization powers, this study aimed reporting 5-ASA solubility in molarity terms in neat ChCl/EG DES (in a molar ratio 1:2) (viscosity of DES is about 48.59 mPa s at 298.15 K [25]), water and their pseudo-binary mixtures in DES mass fractions from 0.1 to 0.9 employing a shake flask way at 293.2–313.2 K and 85 kPa. The molarity of 5-ASA were also represented by some cosolvency models including van’t Hoff [26], mixture response surface (*MRS*) [27], Jouyban-Acree [28], Jouyban-Acree-van’t Hoff [29], the modified version of Jouyban-Acree-van’t Hoff [30, 31], the combined nearly ideal binary solvent/Redlich–Kister (CNIBS/R-K) [32], $\lambda h$ equation [33, 34], the modified Wilson and modified Wilson-van’t Hoff [35]. Aiming to report a main driving force of 5-ASA dissolution process in the above-mentioned mixtures the modified van't Hoff and Gibbs equations [36, 37] were utilized to compute the apparent thermodynamic parameters. The outcomes of this study can expand the available data for 5-ASA solubility in aqueous binary mixtures.

## Materials and methods

### Materials

5-ASA, EG and ChCl were the used materials in present study which a brief summary of their purities and chemical structures, sources are provided in Table 1 along with the more details regarding ethanol and deionized water utilized for diluting the saturated solutions before spectrophotometric experiments.

**Table 1:** Some details of the purity and chemical structure of the employed materials

Material	Mass fraction purity	Source	Chemical formula	Molar mass/g mol^−1^	Structure
Mesalazine (5-ASA)	> 0.999	Julian Khimia Sanat, Iran	C_7_H_7_NO_3_	153.135	
Ethylene glycol (EG)	> 0.999	Merck	C_2_H_6_O_2_	62.07	
Choline chloride (ChCl)	> 0.999	Daejung, Korea	C_5_H_14_NClO	139.62	
Ethanol	0.935	Jahan Alcohol Teb, Arak, Iran	C_2_H_6_O	46.07	
Deionized water		Made in our laboratory	H_2_O	18.02	

The purity of the employed chemicals was provided by the suppliers

ChCl/EG DES was prepared in a molar ratio of 1:2 by mixixng a certain amount of ChCl and EG, as described with details in our previous work [38].

### Solubility determination

The shake-flask method was used to determine the solubility of 5-ASA in ChCl/EG DES (in a molar ratio 1:2), water and ChCl/EG DES + water mixtures. In this respect, an extra amount of 5-ASA was added to tubes containing mono-solvents or binary mixed solvents (with a DES mass fraction of 0.1–0.9 prepared with a digital analytical balance (precision 0.0001 g, Shimadzu, 321-34553, Shimadzu Co., Japan). Then, the mixtures were shacked with a shaking speed of 85 rpm by shaker (rotator 2002, Behdad, Tehran, Iran) placed into incubator equipped with a temperature controlling system with the uncertainty of 0.1 K (Nabziran Industrial Group, Tabriz, Iran) for 48 h to attain an equilibrium, as previously reported in Refs. [9, 20]. After the equilibration, the drug precipitate was separated using centrifugation (Hettich D-7200 centrifuge) to collect the saturated solution followed then by diluting the obtained solutions with water: ethanol mixture (1:1) and assayed by a UV–visible spectrophotometer (Shimadzu UV-1800, Kyoto, Japan) at 299 nm. The concentrations of the diluted solutions in terms of molarity (*C*_1, T_*)* were achieved through the calibration curve (Absorbance = 2911*C*_1, *T*_ + 0.016). A mean value was taken from three experiments at 293.2–313.2 K.

### X-ray powder diffraction

For characterizing the solid phase equilibrated with water, neat ChCl/EG DES in compared with the raw 5-ASA, X-ray powder diffraction (XRD) analysis was utilized. To collect the XRD patterns a Siemens D500 X-ray diffractometer (Germany, Cu Kα radiation (λ = 1.54 A°), 2θ = 10–55°) was utilized. To perform this analysis the excess amounts of 5-ASA were equilibrated with mono-solvents of ChCl/EG DES and water at the same conditions described in the previous subsection. Then, these supernatant mixtures were centrifuged at 10,000 rpm for 30 min to separate the solid drug phase from the mixtures followed by washing the solid drug phases with distilled deionized water for three times and eventually dried at 298.15 K for 96 h.

### Solubility models

The experimental molar solubilities of 5-ASA (*C*_1,T_) in ChCl/EG DES + water were represented utilizing some cosolvency models involving van’t Hoff [26], MRS [27], Jouyban-Acree [28], Jouyban-Acree-van’t Hoff [29], the modified version of Jouyban-Acree-van’t Hoff [30, 31], CNIBS/R-K [32–34], the modified Wilson and modified Wilson-van’t Hoff [35]. Descriptions of these models are provided in the following text:

The Jouyban-Acree model Eq. (1) correlates the drug solubility as a function of temperature and compositions [28].

1 $$\ln C_{{_{1,T} }} = \,w_{2} \ln C_{2,T} + w_{3} \ln C_{3,T} + \frac{{w_{2} w_{3} }}{T}\sum\limits_{i = 0}^{2} {J_{i} .(w_{2} - w_{3} )^{i} }$$

Aiming to provide a more comprehensive model for representing the solubility of drugs in binary mixtures, Eq. (1) can combine with the van’t Hoff equation Eq. (2) [26] as Eq. (3) called the Jouyban-Acree-van’t Hoff model [29].

2 $$\ln C_{{_{1,T} }} = A_{{{2 }or 3}} + \frac{{B_{2 \, or 3} }}{T}$$

3 $$\ln C_{{_{1,T} }} = \,w_{2} \left( {A_{2} + \frac{{B_{2} }}{T}} \right) + w_{3} \left( {A_{3} + \frac{{B_{3} }}{T}} \right) + \frac{{w_{2} w_{3} }}{T}\sum\limits_{i = 0}^{2} {J_{i} .(w_{2} - w_{3} )^{i} }$$

in these equations, $C_{{_{1,T} }}$, $C_{2,T}$ and $C_{3,T}$ denote the molarity of 5-ASA in ChCl/EG DES + water, neat ChCl/EG DES and water, respectively, at temperature *T*/K. *J*_i_ terms were obtained from a linear regression of $\ln C_{{_{1,T} }} - w_{2} \ln C_{2,T} - w_{3} \ln C_{3,T}$ or $\ln C_{{_{1,T} }} - \left( {w_{2} \left( {A_{2} + \frac{{B_{2} }}{T}} \right) + w_{3} \left( {A_{3} + \frac{{B_{3} }}{T}} \right)} \right)$ against $\frac{{w_{2} w_{3} }}{T}$, $\frac{{w_{2} w_{3} }}{T}(w_{2} - w_{3} )$ and $\frac{{w_{2} w_{3} }}{T}(w_{2} - w_{3} )^{2}$, respectively. $w_{2}$ and $w_{3}$ correspond to the mass fractions of ChCl/EG DES and water in the absence of 5-ASA. *A*_1_, *B*_1_, *A*_2_, *B*_2_ are the parameters of Eq. (2).

Also, the molar values of 5-ASA at different compositions of temperatures were modelled with the modified version of Jouyban-Acree-van’t Hoff model Eq. (4) [30, 31] with* D*_1_ to *D*_7_ as the model parameters.

4 $$\ln C_{{_{1,T} }} = D_{1} + \frac{{D_{2} }}{T} + D_{3} w_{2} + D_{4} \frac{{w_{2} }}{T} + D_{5} \frac{{w_{2}^{2} }}{T} + D_{6} \frac{{w_{2}^{3} }}{T} + D_{7} \frac{{w_{2}^{4} }}{T}$$

To represent the drugs solubility at an isotherm condition the *CNIBS/R-K* equation Eq. (5) [32] and *MRS* model Eq. (6) were employed [27].

5 $$\ln C_{{_{1,T} }} = w_{2} \ln x_{2} + w_{3} \ln x_{3} + w_{2} w_{3} \sum\limits_{i = 0}^{2} {S_{i} .(w_{2} - w_{3} )^{i} }$$

6 $$\ln C_{{_{1,T} }} = \beta_{1} w^{\prime}_{2} + \beta_{2} w^{\prime}_{3} + \beta_{3} \left( {\frac{1}{{w^{\prime}_{2} }}} \right) + \beta_{4} \left( {\frac{1}{{w^{\prime}_{3} }}} \right) + \beta_{5} w^{\prime}_{2} w^{\prime}_{3}$$

where *S*_i_ and also $\beta_{1}$ to $\beta_{5}$ are correspondingly the model parameters of Eqs. (5) and (6). In Eq. (6), the values of $w^{\prime}_{2}$ and $w^{\prime}_{3}$ were archived from $w^{\prime}_{2} = 0.96w_{2} + 0.02$ and $w^{\prime}_{3} = 0.96w_{3} + 0.02$, respectively [27].

The modified Wilson Eq. (7), modified Wilson-van't Hoff Eq. (8) [35] and $\lambda h$ equation Eq. (9) [33, 34, 38] are non-linear models for fitting the experimental molar values of 5-ASA in ChCl/EG DES + water mixtures.

7 $$- \ln C_{{_{1,T} }} = 1 - \frac{{w_{2} \left( {1 + \ln C_{2} } \right)}}{{w_{2} + w_{3} \lambda_{23} }} - \frac{{w_{3} \left( {1 + \ln C_{3} } \right)}}{{w_{2} \lambda_{32} + w_{3} }}$$

8 $$- \ln C_{1,T} = 1 - \frac{{w_{2} \left( {1 + A_{2} + \frac{{B_{2} }}{T}} \right)}}{{w_{2} + w_{3} \lambda_{23} }} - \frac{{w_{3} \left( {1 + A_{3} + \frac{{B_{3} }}{T}} \right)}}{{w_{3} + w_{2} \lambda_{32} }}$$

9 $$\ln \left[ {1 + \frac{{\lambda (1 - C_{{_{1,T} }} )}}{{C_{{_{1,T} }} }}} \right] = \lambda h\left[ {\frac{1}{T} - \frac{1}{{T_{m} }}} \right]$$

in these models,$\lambda_{23}$ and $\lambda_{32}$ are the model parameters of Eqs. (7) and (8) whereas $\lambda$ and $h$ correspond to the model constants of Eq. (9) which obtained through a non-linear least square’s regression.

To evaluate the capability of each model in representing of 5-ASA solubility values in molarity terms, the mean percentage deviation (*MPD*), Eq. (10), is utilized.

10 $$MPD = \frac{100}{N}\sum {\left( {\frac{{\left| {C_{1,T}^{\exp } - C_{1,T}^{cal} \, } \right|}}{{C_{1,T}^{\exp } }}} \right)}$$

where *N* is the number of mixtures considered in each case.

### Thermodynamic properties of dissolution

Thermodynamic properties of 5-ASA dissolved in ChCl/EG DES + water including dissolution enthalpy ($\Delta_{sol} H^{ \circ }$), Gibbs free energy of dissolution ($\Delta_{sol} G^{ \circ }$) and dissolution entropy ($\Delta_{sol} S^{ \circ }$) were obtained from the temperature dependency of molar solubilities to provide some useful information regarding the molecular mechanisms involved in the solution processes. At the harmonic temperature (*T*_hm_ = 303.0 K), the values of $\Delta_{sol} H^{ \circ }$, $\Delta_{sol} G^{ \circ }$ and $\Delta_{sol} S^{ \circ }$ were calculated from the van’t Hoff and Gibbs equations Eqs. (11–14) [36, 37].

11 $$\Delta_{sol} H^\circ = - R\left( {\frac{{\partial \ln C_{1,T} }}{{\partial \left( {1/T} \right)_{p} }}} \right) = - R\left( {\frac{{\partial \ln C_{1,T} }}{{\partial \left[ {\left( {1/T} \right) - \left( {1/T_{hm} } \right)} \right]}}} \right)_{p}$$

12 $$T_{hm} = \frac{N}{{\sum\limits_{i = 1}^{N} {\frac{1}{{T_{i} }}} }}$$

13 $$\Delta_{sol} G^\circ = - RT_{hm} .{\text{int}} ercept$$

14 $$\Delta_{sol} S^\circ = \frac{{\Delta_{sol} H^\circ - \Delta_{sol} G^\circ }}{{T_{hm} }}$$

here *R* is the universal gas constant (8.314 J K^−1^ mol^−1^).

The contributions of enthalpy ($\zeta_{H}^{sol}$) and entropy ($\zeta_{TS}^{sol}$) toward dissolution process follow as [39]:

15 $$\zeta_{H}^{sol} = \frac{{\left| {\Delta_{sol} H^{ \circ } } \right|}}{{\left| {\Delta_{sol} H^{ \circ } } \right| + \left| {T_{hm} \Delta_{sol} S^{ \circ } } \right|}}$$

16 $$\zeta_{TS}^{sol} = \frac{{\left| {T_{hm} \Delta_{sol} S^{ \circ } } \right|}}{{\left| {\Delta_{sol} H^{ \circ } } \right| + \left| {T_{hm} \Delta_{sol} S^{ \circ } } \right|}}$$

## Results and discussions

### XRD results

**Fig. 1:**
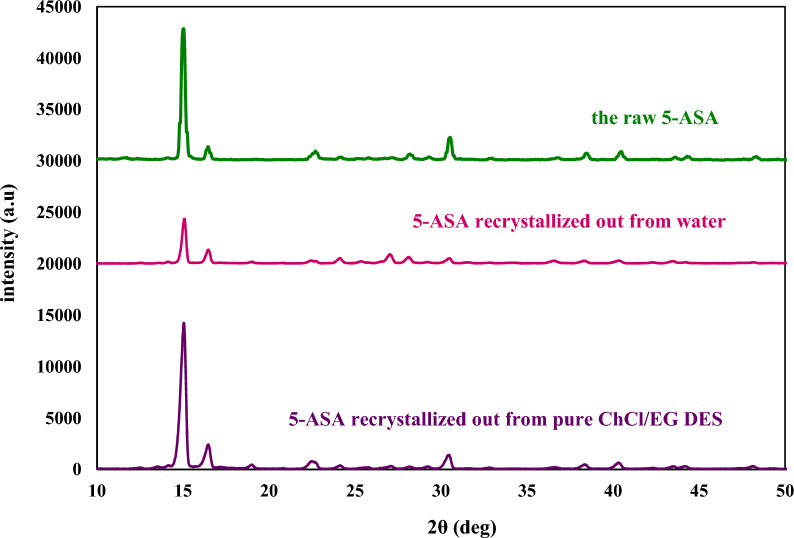
XPRD patterns of the raw 5-ASA and their crystallized out from neat solvents of ChCl/EG DES and water at 298.15 K

The patterns of raw 5-ASA and their equilibrated with liquor are presented in Fig. 1. Based on this figure, all the XRD patterns of solid of 5-ASA in equilibrium with ChCl/EG DES and water have the same characteristic peaks with the raw 5-ASA. Thereby, no polymorph transformation or solvate formation is found during the whole experiment processes.

### Solubility of 5-ASA in aqueous solutions of ChCl/EG DES

**Fig. 2:**
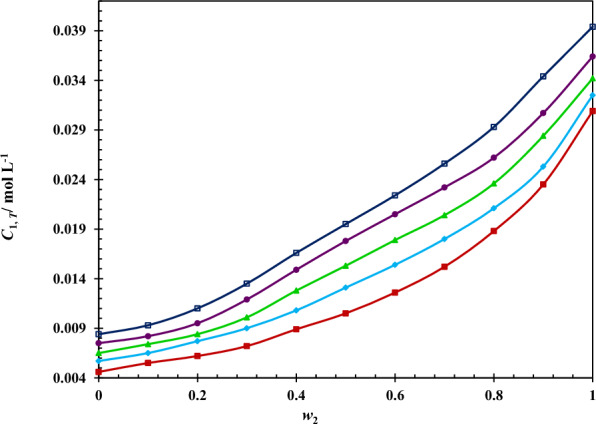
The experimental molar solubility (*C*_1,T_) of 5-ASA in the pseudo-binary solvent mixtures of ChCl/EG DES and water at different temperatures: (), 293.15 K; (), 298.15 K; (), 303.15 K; (), 308.15 K; (), 313.15 K

The experimental molarity of 5-ASA (*C*_1,*T*_) in ChCl/EG DES + water at 293.2–313.2 K are summarized in Table 2 and visually plotted in Fig. 2. As presented in this Table, the solubility of 5-ASA is raised by raising DES mass fraction and also temperature reaching to the highest value in neat ChCl/EG DES at 313.2 K (0.0394 mol L^−1^).

**Table 2:** Experimental solubility of 5-ASA in terms of molarity (*C*_1, *T*_) as the mean of three experiments measured in ChCl/EG DES + water mixtures at 293.2–313.2 K and ambient pressure (≈ 85 kPa)

*w*_2_^*a*^	*C*_1, T_ (mol L^−1^)				
	293.2 K	298.2 K	303.2 K	308.2 K	313.2 K
0.0	0.0046	0.0057	0.0065	0.0075	0.0084
0.1	0.0055	0.0065	0.0074	0.0082	0.0093
0.2	0.0062	0.0077	0.0084	0.0095	0.0110
0.3	0.0072	0.0090	0.0101	0.0119	0.0135
0.4	0.0089	0.0108	0.0128	0.0149	0.0166
0.5	0.0105	0.0131	0.0153	0.0178	0.0195
0.6	0.0126	0.0154	0.0179	0.0205	0.0224
0.7	0.0152	0.0180	0.0204	0.0232	0.0256
0.8	0.0188	0.0211	0.0236	0.0262	0.0293
0.9	0.0235	0.0253	0.0284	0.0307	0.0344
1.0	0.0290	0.0325	0.0342	0.0364	0.0394

Standard uncertainty (*u*) for pressure, temperature and molar concentration of 5-ASA are *u* (*P*) = 0.5 kPa, *u* (*T*) = 0.1 K and u (*C*_1, *T*_) = 0.0150 mol L^−1^, respectively

^*a*^*w*_2_ is mass fraction of ChCl/EG DES in ChCl/EG DES + water mixtures in the absence of 5-ASA with the standard uncertainty (*u*) equal with *u* (*w*_2_) = 0.05

**Fig. 3:**
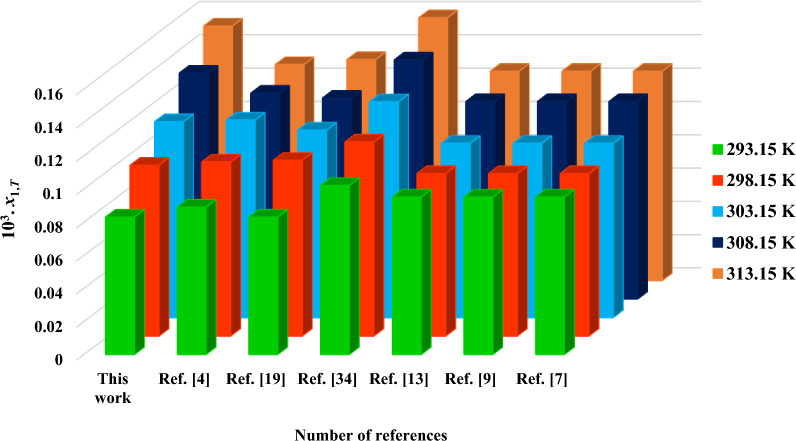
The mole fraction solubility of 5-ASA measured here and the ones reported in the literature [4, 7, 9, 19, 34]

The experimental *C*_1,T_ values in water at 293.2–313.2 K were converted to the mole fraction unite (*x*_1,*T*_) and compared the mole fraction values reported in Refs. [4, 9, 11, 21, 40] to check the accuracy of present data. Table 3 gives the obtained results together with the individual percentage deviations (*IPD*s) computed with Eq. (17).

17 $$IPD = \frac{{{\text{x}}_{1,T}^{\exp } - {\overline{\text{x}}}_{1,T}^{rep} }}{{{\overline{\text{x}}}_{1,T}^{rep} }} \times 100$$

where ${\text{x}}_{1,T}^{\exp }$ and ${\overline{\text{x}}}_{1,T}^{rep}$ are correspondingly the experimental and mean mole fractions of 5-ASA. Also, the mole fraction solubility of 5-ASA determined in this work were compared graphically with the mole fractions reported in the literature [4, 9, 11, 15, 21] and the result is shown in Fig. 3. According to Table 3, the experimental and reported mean values of 10^3^. x_1,T_ in Refs. [4, 9, 11, 15, 21] are 0.083 and 0.095 at 293.2 K; 0.103 and 0.103 at 298.2 K; 0.118 and 0.113 at 303.2 K; 0.136 and 0.125 at 308.2 K and 0.153 and 0.133 at 313.2 K. From Table 3, the minimum and maximum of *IPD*s belong to 293.2 (≈ -14.7%) and 313.2 K (≈ 12.9%), respectively, which these differences may be attributed to the person-to-person error and the employed methodology. Figure 3 and the *IPD*s of Table 3 show that the measured solubility of 5-ASA in present study are not very different from the ones reported in the literature.

**Table 3:** 5-ASA mole fraction solubility (*x*_1, *T*_) data in water along with the corresponding individual percentage deviation (*IPD*%) at various temperatures

	293.2 K	298.2 K	303.2 K	308.2 K	313.2 K	Refs.
10^3^.* x*_1,* T*_	0.083	0.103	0.118	0.136	0.153	This work
	0.089	0.105	0.119	0.124	0.130	[4]
	0.083	0.106	0.113	0.121	0.133	[21]
	0.102	0.117	0.130	0.144	0.158	[40]
	0.095	0.098	0.105	0.119	0.126	[15]
	0.095	0.098	0.105	0.119	0.126	[11]
	0.095	0.098	0.105	0.119	0.126	[9]
Mean of references data	0.095	0.103	0.113	0.125	0.133	
*IPD*%	− 14.7	− 0.2	4.4	8.1	12.9

For the investigation of the influence of cosolvent type on the molarity values of 5-ASA, two defined parameters by Yalkowsky ($\sigma$) [41] Eq. (18) and our group ($\omega$) [8] Eq. (19) were calculated and compared for this drug. Since, the solubility of 5-ASA reported in the literature were in terms of mole fraction, the measured molarity of 5-ASA in this study has been first converted to the mole fraction unit and then utilized in these calculations. For this conversion, the density of ChCl/EG (1.116995 g cm^−3^) and water (0.997050 g cm^−3^) at 298.2 K reported in Ref. [42] was used and the results are summarized in Table 4.

18 $$\sigma = \log \left( {\frac{{x_{1,\cos olvent} }}{{x_{1,water} }}} \right)$$

19 $$\omega = \frac{{\log \left( {\frac{{x_{1,\cos olvent,\max } }}{{x_{1,water} }}} \right)}}{{w_{\cos olvent,\max } }}$$

where $w_{\cos olvent,\max }$ is the mass fraction of cosolvent which achieves the highest 5-ASA solubility. Based on Table 4, the solubility of 5-ASA in the binary mixtures is increased in the order: N-methyl-2-pyrrolidone + water [15] (*σ* = 2.42 and *ω* = 2.42) > betaine/EG + water (*σ* = 2.10 and *ω* = 2.10) [21] > PEG 400 + water [40] (*σ* = 1.84 and *ω* = 1.84) > betaine/PG DES (*σ* = 1.80 and *ω* = 1.80) [21] > ChCl/EG DES (this work) (*σ* = 1.42 and *ω* = 1.42) > EG + water [4] (*σ* = 1.15 and *ω* = 1.15) > PG + water [9] (*σ* = 0.76 and *ω* = 0.91) > ChCl/PG + water [20] (*σ* = 0.70 and *ω* = 0.70) > 1-propanol + water [12] (*σ* = − 0.21 and *ω* = 1.74) ≥ ethanol + water [11] (*σ* = − 0.21 and *ω* = 1.05) > 2-propanol + water [13] (*σ* = − 0.26 and *ω* = 1.71) > acetonitrile + water [14] (*σ* = − 0.90 and *ω* = 1.80). This order shows that the mixtures containing DESs more solubilize 5-ASA in compared with the organic solvents and also the presence of EG as a HBD favors the solubilization of 5-ASA in compared with PG.

**Table 4:** Comparison of the solubilization powers of diversity cosolvents used for 5-ASA

Solvent mixtures	*σ*	*ω*
Choline chloride/ethylene glycol + water	1.42	1.42
Betaine/ethylene glycol + water [21]	2.10	2.10
Betaine/propylene glycol + water [21]	1.80	1.80
Choline chloride/propylene glycol + water [20]	0.70	0.70
Ethylene glycol + water [4]	1.15	1.15
1-propanol + water [12]	− 0.21	1.74
N-methyl-2-pyrrolidone + water [15]	2.42	2.42
Propylene glycol + water [9]	0.76	0.91
Ethanol + water [11]	− 0.21	1.05
2-propanol + water [13]	− 0.26	1.71
Acetonitrile + water [14]	− 0.90	1.80
Polyethylene glycol 400 + water [40]	1.84	1.84

### Solubility modelling

In the following, the solubilities of 5-ASA in ChCl/EG DES + water were correlated with the above-mentioned models and the results are tabulated in Tables 5, 6, 7, 8. In these correlations, the ChCl/EG DES was considered as an associated molecule according to the recent report by Jouyban et al., in regarding with the maintenance of H-bonding ChCl-EG interactions in the most of aqueous binary mixtures (except water mass fractions ≥ 0.7). On the other hand, by considering DES as an associated molecule the number of model’s parameters were also decreased. According to the colected reuslts in Tables 5, 6, 7, 8, the overral *MPD*s (*OMPD*s) of models follow the order: CNIBS/R-K (1.4%) < van’t Hoff (1.6%) < modified Wilson (2.1%) < *MRS* (≤ 2.6) < modified version of Jouyban-Acree-van’t Hoff (3.2%) < Jouyban-Acree (3.7%) < Jouyban-Acree-van’t Hoff (3.9%) < modified Wilson-van’t Hoff (≤ 3.8) < *λh* (5.1%). The low values of *OMPD*s in Tables 5, 6, 7, 8 (≤ 5.1%) prsesnted a good reliability of each model.

**Table 5:** The CNIBS/R-K and modified Wilson model parameters and the corresponding *MPD*% for 5-ASA in ChCl/EG DES + water mixtures

*CNIBS/R-K model*				
	*S*_0_	*S*_1_	*S*_2_	*MPD*%
293.2 K	− 0.395	NS ^*a*^	NS	1.0
298.2 K	− 0.286	NS	NS	1.7
303.2 K	− 0.077	NS	NS	2.6
308.2 K	0.273	0.286	− 1.367	0.8
313.2 K	0.263	0.243	− 0.853	1.0
Overall MPD%				1.4

The modified Wilson model			
	λ_23_	λ_32_	*MPD*%
293.2 K	1.022	0.903	0.9
298.2 K	1.181	0.847	1.7
303.2 K	0.475	1.510	2.3
308.2 K	1.647	0.761	3.5
313.2 K	1.656	0.773	2.2
Overall *MPD*%			2.1

^*a*^ NS denotes to not statistically significant (p-value > 0.05)

**Table 6:** The *λh* and van’t Hoff model parameters and the corresponding *MPD*% for the solubility of 5-ASA in ChCl/EG DES + water mixtures

*w*_2_	*λh model*			van’t Hoff model		
	*λ*	*h*	*MPD*%	*A*	*B*	*MPD*%
0.0	0.503	68.616	0.7	3.927	− 2720.259	1.9
0.1	0.504	67.742	0.7	2.858	− 2358.173	1.2
0.2	0.504	82.819	2.1	3.451	− 2493.718	1.9
0.3	0.505	112.238	1.2	4.728	− 2824.897	1.7
0.4	0.506	0.0001	19.9	5.139	− 2884.878	1.8
0.5	0.508	0.0001	19.6	5.177	− 2843.381	2.8
0.6	0.509	176.462	0.9	4.677	− 2644.598	2.4
0.7	0.510	184.648	0.3	3.963	− 2383.805	1.3
0.8	0.512	183.989	1.1	2.939	− 2026.854	0.7
0.9	0.514	189.917	1.6	2.217	− 1752.760	1.1
1.0	0.517	0.0001	8.6	1.028	− 1334.946	1.0
Overall *MPD*%			5.1			1.6

**Table 7:** The *MRS* model constants at the investigated temperatures and the *MPD*% for back-calculated 5-ASA solubility in ChCl/EG DES + water mixtures

*T*/K	*β*_1_	*β*_*2*_	*β*_3_	*β*_4_	*β*_5_	*MPD*%
293.2	− 3.549	− 5.518	0.002	NS ^*a*^	NS	2.6
298.2	− 3.441	− 5.267	0.001	NS	NS	1.6
303.2	− 3.340	− 5.119	0.001	NS	NS	2.3
308.2	− 3.254	− 4.947	NS	NS	NS	3.9
313.2	− 3.164	− 4.819	NS	NS	NS	2.9
Overall *MPD*%						2.6

^*a*^ NS denotes to not statistically significant (p-value > 0.05)

**Table 8:** The parameters of Jouyban-Acree, Jouyban-Acree-van’t Hoff, the modified version of Jouyban-Acree-van’t Hoff and MW-van’t Hoff models and the corresponding *MPDs*% for 5-ASA in ChCl/EG DES + water mixtures

Jouyban-Acree		Jouyban-Acree-van’t Hoff		Modified version of Jouyban-Acree-van’t Hoff		MW-van’t Hoff	
*J*_0_	− 34.051	*A*_*2*_	1.028	*D*_1_	4.582	*A*_*2*_	1.028
*J*_1_	NS ^*a*^	*B*_*2*_	− 1334.946	*D*_2_	− 2927.627	*B*_*2*_	− 1334.946
*J*_2_	NS	*A*_3_	3.927	*D*_*3*_	− 1.872	*A*_3_	3.927
		*B*_3_	− 2720.259	*D*_4_	1079.206	*B*_3_	− 2720.259
		*J*_0_	− 34.217	*D*_5_	NS	$\lambda_{23}$	1.635
		*J*_1_	NS	*D*_6_	NS	$\lambda_{32}$	0.730
		*J*_2_	NS	*D*_7_	NS		
*MPD*% 3.7		3.9		3.2		3.8	

^*a*^ NS denotes to not statistically significant (p-value > 0.05)

**Fig. 4:**
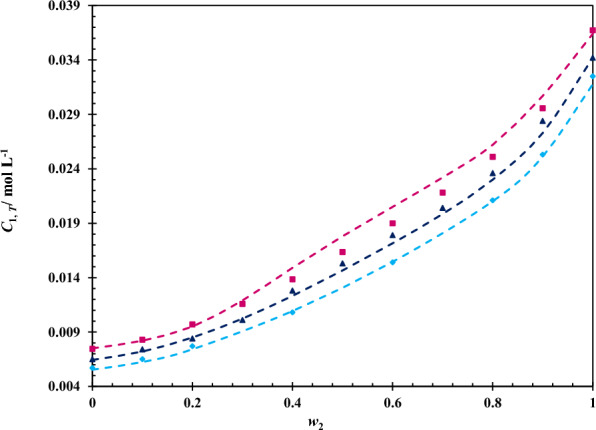
Comparing the experimental values of 5-ASA solubility and those predicted from the Jouyban-Acree-van’t Hoff model with the selection of minimum solubility data in the pseudo-binary mixed solvents of ChCl/EG DES and water: (), 298.2 K; (), 303.2 K; (), 308.2 K; the dash lines obtained from the Jouyban-Acree-van’t Hoff model

Moreover, the minimum number of experimental data, *i.e.* the *C*_1,*T*_ values in mono-solvents of water and ChCl/EG DES at 293.2 and 313.2 K and also in ChCl/EG DES + water mixtures at *w*_2_ = 0.3, 0.5 and 0.7 at 298.2 K were selected and trained the models of Jouyban-Acree, Jouyban-Acree-van’t Hoff and the modified Wilson-van’t Hoff models to obtain the models parameters (see Table 9). The trained models were then utilized to predicted the molarity of 5-ASA in other *w*_2_ at each temperature (*w*_2_ = 0.1 to 0.9 at 293.2 K, 303.2 K, 308.2 K and 313.2 K and also *w*_2_ = 0.0–0.2, 0.4, 0.6, 0.8–1.0 at 298.2 K). From Table 9, it is concluded that the prediction ability of models is acceptable; however, Jouyban-Acree-van’t Hoff model with a lower *MPD*% (≤ 3.9%) is more applicable than the others. A good performance of the Jouyban-Acree-van’t Hoff model in predicting of solubility data can be visually observed by plotting the predicted solubility of 5-ASA from this trained model and the experimental solubility, as seen in Fig. 4.

**Table 9:** The parameters of Jouyban-Acree, Jouyban-Acree-van’t Hoff and modified Wilson-van’t Hoff models along with the corresponding *MPD%* for 5-ASA in the aqueous mixtures of ChCl/EG DES with selection of minimum solubility data, *i.e*. the values of *C*_1,T_ in neat ChCl/EG DES and water at 293.2 K and 313.2 K and DES + water mixtures with *w*_2_ = 0.3, 0.5 and 0.7 at 298.2 K

Jouyban-Acree		Jouyban-Acree-van’t Hoff		the modified Wilson-van’t Hoff	
*J*_0_	− 45.606	*A*_2_	1.028	*A*_2_	1.028
*J*_1_	− 5.612	*B*_2_	− 1334.946	*B*_2_	− 1334.946
*J*_2_	− 309.975	*A*_3_	3.927	*A*_3_	3.927
		*B*_3_	− 2720.259	*B*_3_	− 2720.259
		*J*_0_	− 14.348	$\lambda_{23}$	1.078
		*J*_1_	− 10.289	$\lambda_{32}$	0.928
		*J*_2_	− 272.763		
*MPD*% 4.3		3.9			4.4

### Thermodynamic functions of dissoultion and mixxing of 5-ASA

**Fig. 5:**
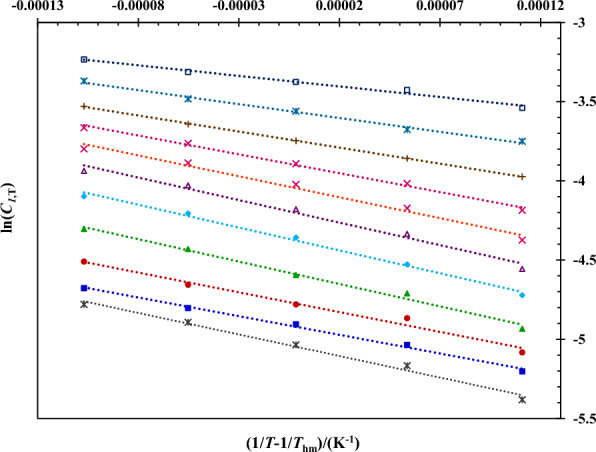
The van’t Hoff plot of logarithm of the molar solubility of 5-ASA in aqueous pseudo-binary mixtures of ChCl/EG DES: (*), *w*_2_ = 0.00 (water); (), *w*_2_ = 0.10; (), *w*_2_ = 0.20; (), *w*_2_ = 0.30; (), *w*_2_ = 0.40; (), *w*_2_ = 0.50; (), *w*_2_ = 0.60; ($\times$), *w*_2_ = 0.70; ( ), *w*_2_ = 0.80; (), *w*_2_ = 0.90; (),* w*_2_ = 1.00 (neat ChCl/EG DES)

The apparent thermodynamic parameters of 5-ASA dissolution in ChCl/EG DES + water mixtures including Δ_sol_*G*°, Δ_sol_*H*° and Δ_sol_*S*° were calculated from plotting the ln*C*_1,*T*_ against the 1/*T*-1/*T*_m_ using their slopes and intercepts presented in Fig. 5. According to this Figure, all mixtures presented a linear trend with a negative slope, showing that the solubility of 5-ASA increase by increasing temperature ($\Delta_{sol} H^{ \circ }$ > 0). Table 10 gives the thermodynamic parameters calculated in the studied mixtures. The positive values of parameters for all mixtures illustrated that the solubility of 5-ASA was an endothermic and entropy-driven procedure (higher values of $\Delta_{sol} S^{ \circ }$ in compared with the values of $\Delta_{sol} H^{ \circ }$) in ChCl/EG DES + water. From Table 10, it is clearly obvious that the values of Δ_sol_*G*° ranged from 8.51 to 12.72 kJ mol^−1^ with the lowest and highest values observed in neat ChCl/EG DES and neat water, respectiely, indicating that the dissolution of 5-ASA in ChCl/EG DES + water mixtures was feasibile at neat DES in good agreement with the solubility measurements.

**Table 10:** Apparent thermodynamic parameters for 5-ASA dissolution behavior in ChCl/EG DES (1:2 molar ratio) + water mixtures at the harmonic temperature (303.0 K).

*w*_2_^*a*^	Δ_sol_*G*^◦^ (kJ mol^−1^)	Δ_sol_*H*^◦^ (kJ mol^−1^)	Δ_sol_*S*^◦^ (J mol^−1^ K^−1^)	*T*Δ_sol_*S*^◦^ (kJ mol^−1^)	*ζ*_H_^sol^	*ζ*_TS_^sol^
0.00	12.72	22.60	32.59	9.87	0.696	0.304
0.10	12.41	19.59	23.70	7.18	0.732	0.268
0.20	12.04	20.73	28.69	8.69	0.705	0.295
0.30	11.57	23.50	39.37	11.93	0.663	0.337
0.40	11.04	24.00	42.78	12.96	0.649	0.351
0.50	10.60	23.62	42.99	13.03	0.645	0.355
0.60	10.20	21.97	38.83	11.77	0.651	0.349
0.70	9.84	19.79	32.86	9.96	0.665	0.335
0.80	9.45	16.85	24.43	7.40	0.695	0.305
0.90	8.99	14.58	18.44	5.59	0.723	0.277
1.00	8.51	11.09	8.52	2.58	0.811	0.189

The combined expanded uncertainties *U* are *U*_c_($\Delta_{sol} H^{ \circ }$) = 0.06 $\Delta_{sol} H^{ \circ }$; Uc($\Delta_{sol} G^{ \circ }$) = 0.06 $\Delta_{sol} G^{ \circ }$ and *U*c($\Delta_{sol} S^{ \circ }$) = 0.06 $\Delta_{sol} S^{ \circ }$(0.95 level of confidence)

^*a*^ *w*_2_ is mass fraction of ChCl/EG DES in ChCl/EG DES + water mixtures in the absence of 5-ASA

Table 10 reports also the values of $\zeta_{H}^{sol}$ and $\zeta_{TS}^{sol}$ which computed with Eqs. (15) and (16), respectively. For all mixtures $\zeta_{H}^{sol}$ > $\zeta_{TS}^{sol}$ which shows that the enthalpy is the main contributor of $\Delta_{sol} G^{ \circ }$ in 5-ASA dissolution process.

**Fig. 6:**
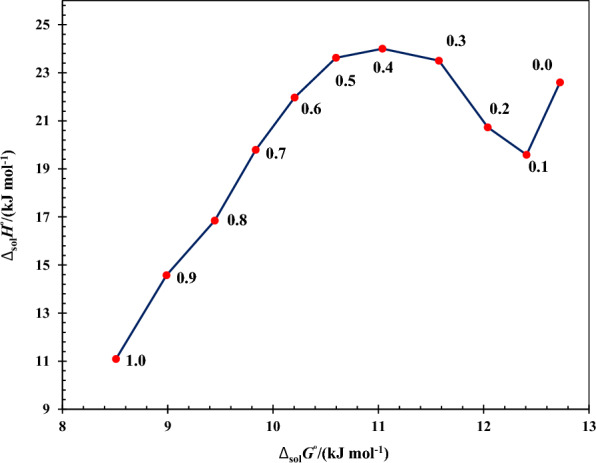
Enthalpy-entropy compensation plot for 5-ASA in the aqueous pseudo-binary mixtures of ChCl/EG DES at *T*_hm_ = 303.0  K. The points present the mass fraction of ChCl/EG DES in the investigated mixtures in the absence of 5-ASA

The enthalpy-entropy compensation analysis were carried out and the result is plotted in Fig. 6. From this Figure, the non-linear trend observed with the negative slopes in 0.0 ≤ *w*_2_ ≤ 0.3 and 0.4 ≤ *w*_2_ ≤ 1.0 along with the positive slopes in the other compositions. The negative and positive slopes in this plot presented that the driving mechanism is the entropy as a results of hydrophobic hydration around the non-polar groups of 5-ASA and enthalpy as a consequent of better solvation of 5-ASA by the cosolvent, respectively.

## Conclusions

Equilibrium solubility of mesalazine (5-ASA) in pseudo-binary mixed solvents of choline chloride/ethylene glycol deep eutectic solvent (ChCl/EG DES, molar ratio of 1:2) and water were measured at 293.2–313.2 and ambient pressure. After that, the experimental solubility was modelled and the solubility data were back-computed with the cosolvency models including van’t Hoff, mixture response surface (*MRS*), Jouyban-Acree, Jouyban-Acree-van’t Hoff, the modified version of Jouyban-Acree-van’t Hoff, the combined nearly ideal binary solvent/Redlich–Kister (CNIBS/R-K), $\lambda h$ equation, the modified Wilson and modified Wilson-van’t Hoff. The following results were obtained from this study:

- From surveying the solubility profile of 5-ASA, a solvent composition and temperature dependency was observed; so that, more solubilization of 5-ASA achieved in high mass fraction of DES and temperature (0.0394 mol L^−1^ at 313.2 K).
- From investigating the thermodynamic parameters of 5-ASA dissolution process in the investigated mixtures, observed an entropy-driven and endothermic process with the lowest Gibbs free energy in neat ChCl/EG DES.
- According to the low mean percentage deviation of models (≤ 5.14%) for the back-computed data with the cosolvency models, an acceptable ability of the models for modelling of 5-ASA solubility was confirmed.

The measured data in this study not only expanded the solubility database of 5-ASA in cosolvency mixtures but also can be used for the selection of a suitable mono- or binary solvent mixtures to purify of 5-ASA through the crystallization.

## Data Availability

The datasets utilized and/or analyzed during this study are available from the corresponding author on reasonable request.

## Abbreviations

5-ASA: Mesalazine
ChCl/EG DES: Choline chloride/ethylene glycol deep eutectic solvent
XRD: X-ray powder diffraction
PG: Propylene glycol
PEG: 400 Polyethylene glycol 400
EG: Ethylene glycol
PEGDME 250: Polyethylene glycol dimethyl ether 250
CNIBS/R-K: The combined nearly ideal binary solvent/Redlich–Kister
MPD: Mean percentage deviation
IPDs: Individual percentage deviations
OMPDs: Overall mean percentage deviation

## References

[1] Brogden RN, Sorkin EM (1989). Mesalazine: a review of its pharmacodynamic and pharmacokinetic properties, and therapeutic potential in chronic inflammatory bowel disease. Drugs.

[2] Patel A, Vaghasiya A, Gajera R, Baluja S (2010). Solubility of 5-amino salicylic acid in different solvents at various temperatures. J Chem Eng Data.

[3] Jouyban A (2010). Handbook of solubility data for pharmaceuticals.

[4] Sheikhi-Sovari A, Jouyban A, Martinez F, Hemmati S, Rahimpour E (2021). Solubility of mesalazine in ethylene glycol+ water mixtures at different temperatures. J Mol Liq.

[5] Lau ETL, Giddings SJ, Mohammed SG, Dubois P (2013). Encapsulation of hydrocortisone and mesalazine in zein microparticles. Pharm.

[6] Pawar AR, Mundhe PV, Deshmukh VK, Pandhare RB, Nandgude TD (2021). Enrichment of aqueous solubility and dissolution profile of mesalamine: In vitro evaluation of solid dispersion and employing complexing agents. J Pharm Bio Sci.

[7] Kumar A, Sahoo SK, Padhee K, Kochar PS, Sathapathy A, Pathak N (2011). Review on solubility enhancement techniques for hydrophobic drugs. Pharmacie Globale.

[8] Jouyban A (2019). Review of the cosolvency models for predicting drug solubility in solvent mixtures: an update. J Pharm Pharma Sci.

[9] Jouyban-Gharamaleki V, Rahimpour E, Hemmati S, Martinez F, Jouyban A (2020). Mesalazine solubility in propylene glycol and water mixtures at various temperatures using a laser monitoring technique. J Mol Liq.

[10] Moradi M, Rahimpour E, Hemmati S, Martinez F, Barzegar-Jalali M, Jouyban A (2020). Solubility of mesalazine in polyethylene glycol 400+ water mixtures at different temperatures. J Mol Liq.

[11] Alvani-Alamdari S, Rezaei H, Rahimpour E, Hemmati S, Martinez F, Barzegar-Jalali M, Jouyban A (2021). Mesalazine solubility in the binary mixtures of ethanol and water at various temperatures. Phys Chem Liq.

[12] Mazaher-Haji Agha E, Barzegar-Jalali M, Adibkia K, Hemmati S, Kuentz M, Martinez F, Jouyban A (2020). Solubility of mesalazine in 1-propanol/water mixtures at different temperatures. J Mol Liq.

[13] Mazaher-Haji Agha E, Barzegar-Jalali M, Adibkia K, Hemmati S, Martinez F, Jouyban A (2020). Solubility and thermodynamic properties of mesalazine in 2-propanol+ water mixtures at various temperatures. J Mol Liq.

[14] Barzegar-Jalali M, Mazaher-Haji Agha E, Adibkia K, Hemmati S, Martinez F, Jouyban A (2021). Solubility of mesalazine in acetonitrile+ water mixtures at various temperatures. Phys Chem Liq.

[15] Jouyban K, Mazaher-Haji Agha E, Hemmati S, Martinez F, Kuentz M, Jouyban A (2020). Solubility of 5-aminosalicylic acid in N-methyl-2-pyrrolidone + water mixtures at various temperatures. J Mol Liq.

[16] Barzegar-Jalali M, Jafari P, Hemmati S, Jouyban A (2023). Equilibrium solubility investigation and thermodynamic aspects of paracetamol, salicylic acid and 5-aminosalicylic acid in polyethylene glycol dimethyl ether 250+ water mixtures. J Mol Liq.

[17] Moradi M, Mazaher-Haji Agha E, Hemmati S, Martinez F, Kuentz M, Jouyban A (2020). Solubility of 5-aminosalicylic acid in N-methyl-2-pyrrolidone + ethanol mixtures at T=(293.2 to 313.2) K. J Mol Liq..

[18] Rezaei H, Jouyban A, Martinez F, Barzegar-Jalali M, Hemmati S, Rahimpour E (2021). Solubility and thermodynamic profile of mesalazine in carbitol + ethanol mixtures at different temperatures. J Mol Liq.

[19] Anastas P, Eghbali N (2010). Green Chemistry principle and practice. Chem Soc Rev.

[20] Zarghampour A, Moradi M, Martinez F, Hemmati S, Rahimpour E, Jouyban A (2021). Solubility study of mesalazine in the aqueous mixtures of a deep-eutectic solvent at different temperatures. J Mol Liq.

[21] Jafari P, Barzegar-Jalali M, Jouyban A (2022). Solubility of mesalazine in aqueous solutions of two betaine-based deep eutectic solvents at different temperatures: data correlation and thermodynamic analysis. J Mol Liq.

[22] Shekaari H, Zafarani-Moattar MT, Mokhtarpour M, Faraji S (2019). Exploring cytotoxicity of some choline-based deep eutectic solvents and their effect on the solubility of lamotrigine in aqueous media. J Mol Liq.

[23] Shekaari H, Zafarani-Moattar MT, Shayanfar A, Mokhtarpour M (2018). Effect of choline chloride/ethylene glycol or glycerol as deep eutectic solvents on the solubility and thermodynamic properties of acetaminophen. J Mol Liq.

[24] Shekaari H, Zafarani-Moattar MT, Mokhtarpour M, Faraji S (2023). Solubility of hesperidien drug in aqueous biodegradible acidic choline chloride-based deep eutectic solvents. Sci Rep.

[25] Gajerdo-Parra NF, Cotroneo-Figueroa VP, Aravena P, Vesovic V, Canales RI (2020). Viscosity of choline chloride-based deep eutectic solvents: Experiments and modeling. J Chem Eng Data.

[26] Hatefi A, Jouyban A, Mohammadian E, Acree WE, Rahimpour E (2019). Prediction of paracetamol solubility in cosolvency systems at different temperatures. J Mol Liq.

[27] Ochsner AB, Belloto RJ, Sokoloski TD (1985). Prediction of xanthine solubilities using statistical techniques. J Pharm Sci.

[28] Jouyban A, Acree WE (2018). Mathematical derivation of the Jouyban-Acree model to represent solute solubility data in mixed solvents at various temperatures. J Mol Liq.

[29] Fathi-Azarbayjani A, Abbasi M, Vaez-Gharamaleki J, Jouyban A (2016). Measurement and correlation of deferiprone solubility: Investigation of solubility parameter and application of van't Hoff equation and Jouyban-Acree model. J Mol Liq.

[30] Ma H, Qu Y, Zhou Z, Wang S, Li L (2012). Solubility of thiotriazinone in binary solvent mixtures of water + methanol and water + ethanol from (283 to 330) K. J Chem Eng Data.

[31] Gao Q, Zhu P, Zhao H, Farajtabar A, Jouyban A, Acree WE (2021). Solubility, Hansen solubility parameter, solvent effect and preferential solvation of benorilate in aqueous mixtures of isopropanol, N, N-dimethylformamide, ethanol and N-methyl-2-pyrrolidinone. J Chem Thermodyn.

[32] Acree WE (1992). Mathematical representation of thermodynamic properties: Part 2. Derivation of the combined nearly ideal binary solvent (NIBS)/Redlich-Kister mathematical representation from a two-body and three-body interactional mixing model. Thermochim Acta.

[33] Buchowski H, Khiat A (1986). Solubility of solids in liquids: one-parameter solubility equation. Fluid Phase Equilib.

[34] Buchowski H, Ksiazczak A, Pietrzyk S (1980). Solvent activity along a saturation line and solubility of hydrogen-bonding solids. J Phys Chem.

[35] Jouyban-Gharamaleki A (1998). The modified wilson model and predicting drug solubility in water-cosolvent mixtures. Chem Pharm Bull.

[36] Wu YF, Cheng L, Wu JQ, Zhai MY, Cai LE (2019). Cosolvent effect on solubility of aripiprazole in mixed solvents and apparent thermodynamic analysis of dissolution process. J Chem Thermodyn.

[37] Guoquan Z (2021). The dissolution behavior and thermodynamic properties of amlodipine in mixtures of n-propanol/isopropanol + water. J Chem Thermodyn.

[38] Moradi M, Jouyban A (2022). Study of naproxen dissolution in the mixtures of a choline-based deep eutectic solvent + water at different temperatures. J Mol Liq.

[39] Aragon DM, Eduardo RJ, Martinez F (2010). Thermodynamic study of the solubility of ibuprofen in acetone and dichloromethane. Braz J Pharm Sci.

[40] Rahimpour E, Moradi M, Hemmati S, Martinez F, Barzegar-Jalali M, Jouyban A (2020). Solubility of mesalazine in polyethylene glycol 400 + water mixtures at different temperatures. J Mol Liq.

[41] Li A, Yalkowsky SH (1998). Cosolvency 1, Solubility Ratio and Solute log Kow, Ind. Eng Chem Res.

[42] Wang Y, Ma C, Liu C, Lu X, Feng X, Ji X (2020). Thermodynamic study of choline chloride-based deep eutectc solvents with water and methanol. J Chem Eng Data.

